# Decreased Global Interest in Oral Cancer During the COVID-19 Pandemic

**DOI:** 10.31557/APJCP.2021.22.7.2117

**Published:** 2021-07

**Authors:** Junhel Dalanon, Yoshizo Matsuka

**Affiliations:** 1 *School of Dentistry, Southwestern University PHINMA, Cebu City, Cebu, Philippines.*; 2 *Department of Stomatognathic Function and Occlusal Reconstruction, Tokushima University Graduate School of Biomedical Sciences, Tokushima City, Tokushima, Japan.*

**Keywords:** Cancer prevention, COVID-19, health-seeking behavior, oral cancer, trend analysis

## Abstract

**Objective:**

Oral cancer is one of the most common malignancies in developing countries, but studies using global data are scarce. The aim of this study is to analyze the search interests for oral cancer using mouth cancer, tongue cancer, gum cancer, and lip cancer as common keywords.

**Methods:**

Internet searches relating to oral cancer from 2010 to 2020, from 250 countries and dependent areas, were retrieved from Google Trends. Color densities in a heat map were used to show geographic differences. Kruskal-Wallis test with post hoc Dunn’s analysis was used to perform yearly comparisons of searches for mouth cancer, tongue cancer, gum cancer, and lip cancer. Search results within 2020 were also compared to determine differences. Forecasting searches from 2021 to 2022 were done by fitting time series models.

**Results:**

From 29 of 250 (11.6%) countries, the highest search values were observed for mouth cancer in Sri Lanka, Qatar, Bangladesh, Finland, Netherlands, Spain, and France. Compared to 2020, greater searches were seen in 2018 (Mdn = 91%, P = 0.023) and 2019 (Mdn = 94%, P = 0.012) for mouth cancer, and 2019 (Mdn = 17%, P = 0.035) for lip cancer. The relative search volumes for gum cancer and lip cancer were substantially lower than mouth cancer during the COVID-19 pandemic.

**Conclusion:**

Higher-income countries tend to be more interested in seeking information about oral cancer. Noteworthy decline in the interest in seeking information online for oral cancer may have crucial implications during the COVID-19 pandemic. Google Trends offer an invaluable and inexpensive means for oral cancer surveillance and health-seeking behavior.

## Introduction

Worldwide, oral cancer is the 15^th^ leading cause of death and 16^th^ most common malignancy. The oral cancer incidence of four per 100, 000 cases vary depending on countries, races, age, gender, and socioeconomic situations (Ferlay et al., 2019). The risk factors for oral cancer include genetic and environmental factors, and physical conditions. Discrepancies per country could be due to preventive education, culture, medical archiving conditions, and life expectancies (Wyss et al., 2013). Roughly 657, 000 new instances and 330, 000 subsequent deaths due to oral cancer have been reported worldwide each year. Betel nut chewing, excessive drinking, and smoking are health-related behaviors that could lead to heightened risk of oral cancer occurrence. Although socioeconomic status has been emphasized and linked to increased oral cancer incidence, it has also been shown that highly urbanized areas had have greater risk for cancer as well (Hung et al., 2020).

Early cancer prevention and detection is a vital component in the control of cancer. Expensive and highly invasive treatment modalities can be avoided through early discovery of cancer (Sankaranarayanan and Boffetta, 2010). Currently, Google Trends (GT) enable cost-effective surveillance of various diseases and other health-related phenomena. Among others, scientists and epidemiologists have used GT in the past to detect health-seeking behavior in terms of toothache (Dalanon et al., 2021), tooth decay (Dalanon and Matsuka, 2020b; Dalanon and Matsuka, 2020a), health professions (Dalanon and Matsuka, 2020c; Dalanon and Matsuka, 2020d), influenza (Choi and Ahn, 2020), Zika (Strauss et al., 2020), and COVID-19 (Adkins and Booth, 2020; Sycinska-Dziarnowska and Paradowska-Stankiewicz, 2020). A previous study analyzed search interests for oral cancer in Filipino people, but an analysis of global GT data has not been done yet (Dalanon et al., 2020). In addition, the existing contagion brought about by the SARS-CoV-2 (Severe acute respiratory syndrome coronavirus 2) may have implications in cancer prevention and treatment in Asian and low- and middle-income countries (Gatellier et al., 2021; Somathunga et al., 2021; Varela-Centelles et al., 2021; Yadav et al., 2021).

Despite what has currently been known about oral cancer, there is still a lack of literature about oral cancer. To the knowledge of the researchers, there has been no published study that performed surveillance of oral cancer using global data from GT and taking in to account 250 countries and dependent areas. This study aimed to analyze the world searches for oral cancer. Specifically, this research was geared towards (1) comparing the geographic variances in searches for oral cancer from 2010 to 2020; (2) evaluating the timeline differences in oral cancer online queries; and (3) forecasting search trends from 2021 to 2022 through model-fitting.

## Materials and Methods

*Data Search and Retrieval*

The search terms “mouth cancer” (MC), “tongue cancer” (TC), “gum cancer” (GC), and “lip cancer” (LC) under the category of “health”, “web search” as database, from January 2010 to December 2020, and “worldwide” as location were used in GT as search parameters (https://bit.ly/3x3VN3c). Graphs generated by the graphic user interface were used to initially assess geographic and timepoint differences. These included the subregion comparison breakdown chart, interest over time chart, related queries per search term, and interest by subregion per search term data. GT uses relative search volume (RSV) in units labeled peak popularity that is expressed in percentages ranging from a minimum of 0 to a maximum of 100. The minimum value is plotted when data is absent or inadequate. These RSV data were compiled in Comma Separated Value (CSV) files. The CSV files for subregion comparison or comparison across countries and interest over time were downloaded to a spreadsheet software.

*Analyses for Geographic and Timeline Differences*

The RSV form the CSV file on countries comparison were used to create a heatmap on a map chart. The color intensity in the map is descriptive of the varying levels of search interests per search term. A nonparametric two-way Kruskal-Wallis test was used to compare the mean rank of each year from 2010 to 2019 with the mean rank of 2020 as the control year for each search term. Dunn’s multiple comparison test with multiplicity adjusted P-value for each comparison at 0.05 confidence level was then used for hypothesis testing. The same statistical testing procedure was used to compare the RSV of TC, GC, and LC with MC across months within 2020. All inferential statistics were done in GraphPad Prism (version 9).

*Time Series Modelling and Forecasting*

The time series module of IBM SPSS (version 26) is used for forecasting by quantifying a distinct variable repeatedly across time. The obtained data from observations are then used to forecast forthcoming occurrences. Model-fitting and automatic identification of models were done through the Expert Modeler feature. Simple seasonal model was consequentially used as there was no seasonal trend and effect seen in the dataset for all search terms.

## Results

*Geographical Differences of Internet Searches for Oral Cancer*

**Figure 1:**
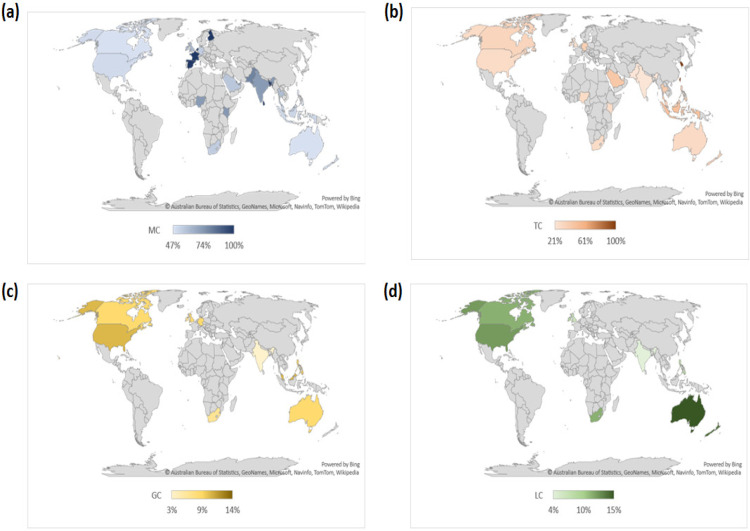
Heat Map Showing Geographic Differences of Internet Searches for Oral Cancer (n=37). More searches for (a) mouth cancer were observed in Sri Lanka (100%), Qatar (100%), Bangladesh (100%), Finland (100%), Netherlands (100%), Spain (100%), and France (100%). While increased search volumes for (b) tongue cancer were found in Hong Kong (100%), South Korea (100%), and Taiwan (100%). Although not as great as the former two sites of oral cancer, the searches for (c) gum cancer were more pronounced in the countries of Singapore (14%), Malaysia (10%), and United States of America (10%). Similarly, the searches for lip cancer yielded lower search volumes but peaks were higher in Australia (15%), New Zealand (14%), and the United States of America (12%)

About 29 out of 250 countries yielded computable RSVs. Roughly 18 are high-income countries, 4 from upper middle-income countries, and 7 from lower middle-income countries (Table 1). The generated map charts showed Sri Lanka (100%), Qatar (100%), Bangladesh (100%), Finland (100%), Netherlands (100%), Spain (100%), and France (100%) were the countries with the highest RSV for MC from January 2010 to December 2020 (Figure 1a). Additionally, Hong Kong (100%), South Korea (100%), and Taiwan (100%) showed the most searches for TC (Figure 1b). Moreover, Singapore (14%), Malaysia (10%), and the United States of America (10%) were the countries with the most searches for GC (Figure 1c). Lastly, the top three countries with the highest searches for LC were Australia (15%), New Zealand (14%), and the United States of America (12%) (Figure 1d). Despite the results, there were no significant correlations found between high-income versus middle-income countries in terms of MC (Rs = -0.026, P = 0.941), TC (Rs = 0.112, P = 0.739), GC (Rs = -0.247, P = 0.512), and LC (Rs = -0.141, P = 0.661) (Table 2).

*Timeline Differences in the Global Search for Oral Cancer*

**Figure 2:**
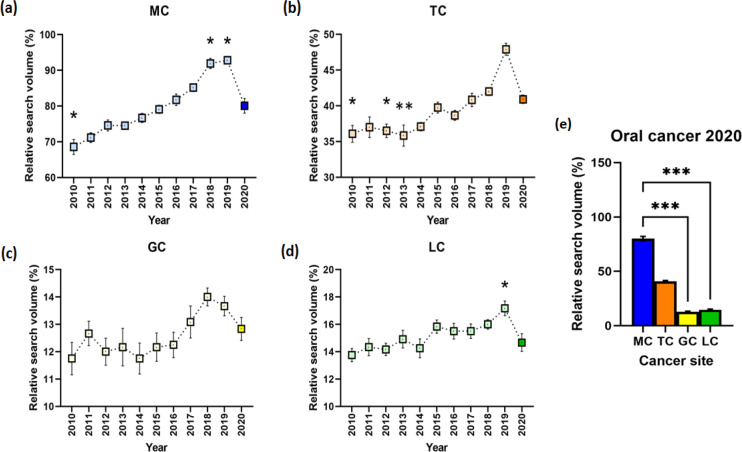
Timeline Differences in the Global Search for Oral Cancer (n=12). The relative search volume (RSV) for (a) mouth cancer (MC) was found to be substantially lesser in 2010, but greater in 2018 and 2019, when compared to 2020. The RSV for (b) tongue cancer (TC) was significantly reduced in 2010, 2012, and 2013 compared to 2020 but no noteworthy disparity was observed in (c) gum cancer (GC). However, considerably greater searches for (d) lip cancer (LC) can be seen in 2019 compared with 2020. (e) In 2020 when the COVID-19 pandemic was widespread, the RSV for GC and LC were significantly lower compared to MC, but no difference was found with TC. *p < 0.05, **p < 0.01, ***p < 0.001

Substantial differences were seen in the search for MC, H (11) = 90.1, P < 0.001. The RSV for 2010 (Mdn = 67%, P = 0.030) was relatively lower compared to 2020 (Mdn = 79.5). However, noteworthy increases were seen in 2018 (Mdn = 91%, P = 0.023) and 2019 (Mdn = 94%, P = 0.012) (Figure 2a). Moreover, searches for TC yielded significant difference when yearly searches from 2010-2019 were compared with 2020, H (11) = 66.78, P < 0.001. It can be noted that the RSV on 2010 (Mdn = 37%, P = 0.041), 2012 (Mdn = 35.5%, P = 0.042), and 2013 (Mdn = 33.5%, P = 0.007) were significantly lower compared to 2020 (Mdn = 41%) (Figure 2b). Similarly, the comparison of RSV for GC yielded remarkable variances, H (11) = 21.17, P = 0.020. However, the post hoc Dunn’s test did not yield any difference when years were compared with 2020 (Figure 2c). In contrast, significant differences in the searches for LC were found, H (11) = 29.7, P < 0.001. In addition, the RSV in 2019 (Mdn = 17%, P = 0.035) was relatively higher compared to 2020 (Mdn = 14%) (Figure 2d).

Comparing the health-seeking behavior through searches relating to oral cancer sites during the COVID-19 pandemic was also a point of interest. In this comparison, searches done in the year 2020 for TC, GC, and LC were compared with MC. Substantial discrepancies were seen upon evaluation, H (4) = 40.9, P < 0.001. Further scrutiny showed that searches for GC (Mdn = 13%, P < 0.001) and LC (Mdn = 14%, P < 0.001) were substantively lower than MC (Mdn = 79.5%). However, no difference was found when the RSV for TC (Mdn = 41%, P = 0.105) was compared with MC (Figure 2e).

*Forecasting Oral Cancer Searches from 2021-2022*

**Figure 3:**
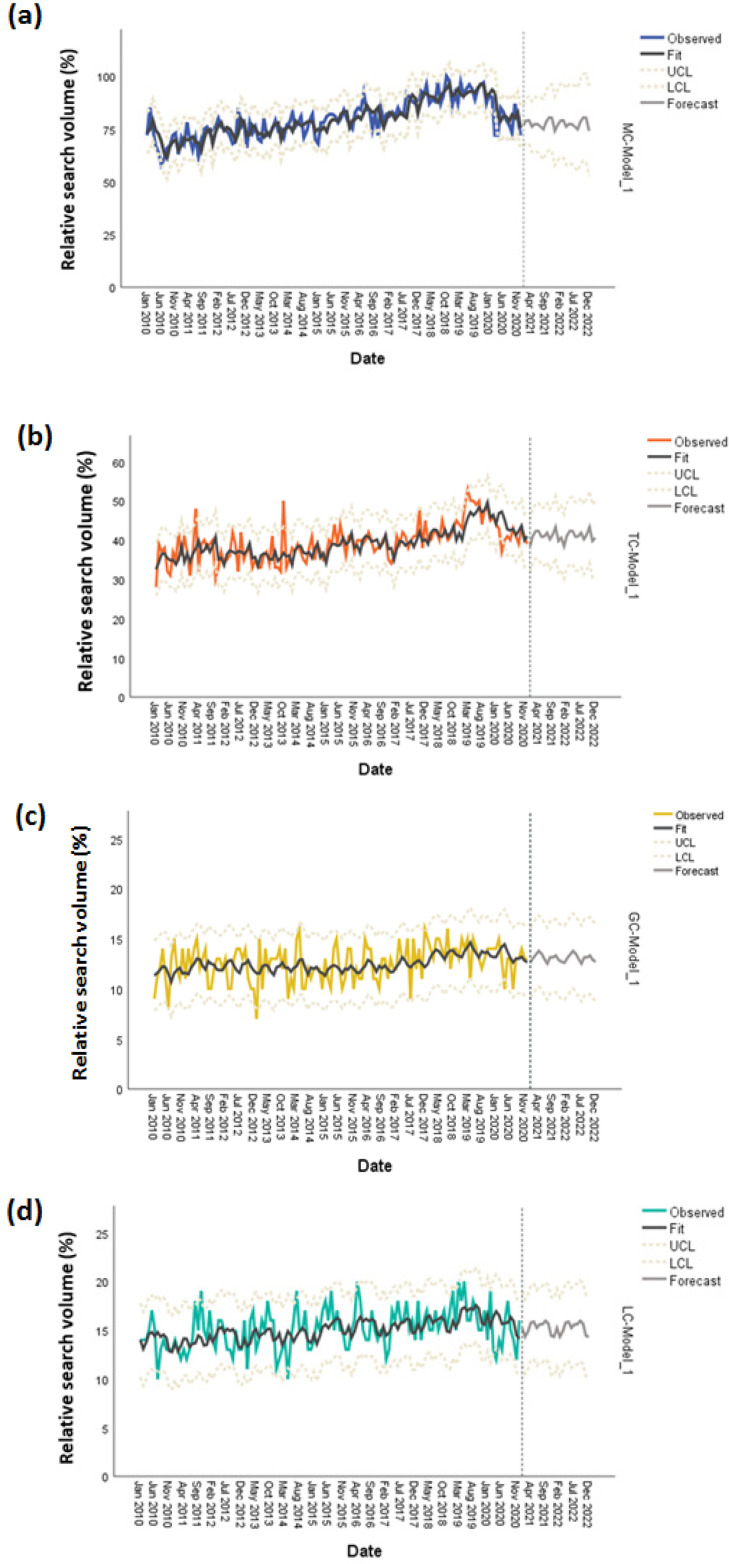
Forecasting Oral Cancer Searches in 2021 and 2022 (n=132). Monthly relative search volume (RSV) was plotted showing the observed values, fit values, upper class limit (UCL), lower class limit (LCL) and forecasted values for (a) mouth cancer, (b) tongue cancer, (c) gum cancer, and (d) lip cancer

As IBM SPSS expert modeler did not find any seasonal trend, the simple seasonal model was fitted in all of the data sets to produce forecasted values. The raw RSV data for MC show that the lowest value was 58% on June 2010 with a peak value of 100% during October 2018. The highest forecasted value in 2021 will be 80% (October and November) and similarly 80% in 2022 (October and November) (Figure 3a). In terms of TC, the lowest RSV of 28% was recorded on January 2010 with an all-time high of 53% on March 2019. An estimated 43% peak popularity can be seen as the highest in 2021 and 2022 during October (Figure 3b). The search for GC had the highest search value of 16% on April 2014, December 2017, and August 2018. The lowest RSV was found on January 2013 (7%). The 14% predicted value will be the highest in 2021 (April and May) and 2022 (April and May) (Figure 3c). With a search value of 20%, the highest search queries were found on April 2016, March 2019, and May 2019. At 10%, the lowest searches were done on July 2010 and April 2014. Multiple timepoints of 16% RSV for LC searches will occur on April, May, July, August, September, and October of 2021 and 2022 (Figure 3d).

**Table 1:** Search Interests of Countries for Oral Cancer Based on Relative Search Volumes

Country	MC	TC	GC	LC	World Bank Classification
Australia	48%	29%	8%	15%	HI
Bangladesh	100%	0%	0%	0%	LMI
Canada	48%	33%	8%	11%	HI
Finland	100%	0%	0%	0%	HI
France	100%	0%	0%	0%	HI
Germany	55%	37%	8%	0%	HI
Hong Kong	0%	100%	0%	0%	HI
India	72%	21%	3%	4%	LMI
Indonesia	52%	48%	0%	0%	UMI
Ireland	61%	25%	6%	8%	HI
Kenya	73%	27%	0%	0%	LMI
Malaysia	56%	34%	10%	0%	UMI
Netherlands	100%	0%	0%	0%	HI
New Zealand	52%	34%	0%	14%	HI
Nigeria	72%	28%	0%	0%	LMI
Pakistan	79%	21%	0%	0%	LMI
Philippines	47%	36%	9%	8%	LMI
Qatar	100%	0%	0%	0%	HI
Saudi Arabia	57%	43%	0%	0%	HI
Singapore	53%	33%	14%	0%	HI
South Africa	55%	28%	6%	11%	UMI
South Korea	0%	100%	0%	0%	HI
Spain	100%	0%	0%	0%	HI
Sri Lanka	100%	0%	0%	0%	LMI
Taiwan	0%	100%	0%	0%	HI
Thailand	60%	40%	0%	0%	UMI
United Arab Emirates	71%	29%	0%	0%	HI
United Kingdom	63%	23%	8%	6%	HI
United States	50%	28%	10%	12%	HI

MC, mouth cancer; TC, tongue cancer; GC, gum cancer; LC, lip cancer; HI, high-income country; LMI, lower middle-income country; UMI, upper middle-income country

**Table 2:** Correlation between the Searches for MC, TC, GC, and LC

	MC	TC	GC	LC
R	-0.02577	0.1124	-0.2469	-0.1406
95% confidence interval	-0.6287 to 0.5965	-0.5374 to 0.6785	-0.7468 to 0.4311	-0.6937 to 0.5167
P (two-tailed)	0.941	0.739	0.512	0.661
P value summary	ns	ns	ns	ns
Exact or approximate P value?	Exact	Exact	Exact	Exact
Significant? (alpha = 0.05)	No	No	No	No

MC, mouth cancer; TC, tongue cancer; GC, gum cancer; LC, lip cancer; ns, no significance

## Discussion

The use of GT is an inexpensive and rapid method of gathering real-time or archived data on internet searches for a particular disease or health occurrences. This study revealed that four out of seven countries that searched highest for MC are European (Finland, Netherlands, Spain, and France) and five are from high-income economies (Qatar, Finland, Netherlands, Spain, and France). Despite belonging to Asia, the three nations (Hong Kong, South Korea, and Taiwan) that searched highest for TC are high-income countries as well (Figure 1a-d). The extent of impact and implementation of advocacy, screening, education, early diagnosis, awareness, legislation, treatment, and vaccination varies across different economic levels of countries. This is an important consideration in determining the health-seeking behavior of a population. In addition, a key challenge for cancer prevention mediation and studies is the transfer of cancer risks across these countries (Sankaranarayanan and Boffetta, 2010). Likewise, internet connectivity and social media analysis can expose underserved people from remote areas who are disjointed from community health services (Yeung, 2018). Previous studies using GT showed that cancer prevention advocacy programs can improve health-seeking behaviors. For instance, Mouth Cancer Awareness Day in Ireland that was launched in September 2010 improved public awareness of oral cancer. This may improve earlier diagnosis and healthier prognosis of cancer cases (Murray et al., 2016). In Malaysia, the breast cancer awareness campaign of Pink October may have spiked interest in breast cancer treatment or screening and may have implications in geographic differences as well (Mohamad and Kok, 2019). The celebration of the Philippine National Oral Health Month with the moniker Purple Feb may be implicated in the rise of oral cancer-related searches during February. Regional differences were also observed, where some urbanized areas may have high incidence of oral cancer due to sex trade. While the heightened cases in the rural areas may be due to the tobacco industry and betel nut chewing (Dalanon et al., 2020).

Similarly, the top three nations that showed interest for GC were from high-income (Singapore and USA) and middle-income countries (Malaysia) (Figure 1c). A study in 1962 in Asia attributed labio-gingival sulcus cancer with the use of khaini and buccal cancer with betel nut chewing. This could explain the increased search queries for GC in Singapore and Malaysia (Muir, 1962). Interestingly, GC is rare and accounts for only 10% of oral cancers in the USA and Europe. In contrast, it is the second most common malignancy in Japan. It can often be asymptomatic and studied together with other oral cancer subsites. This makes detection and diagnosis difficult (Bark et al., 2016).

In this study, all three countries that topped the internet queries for LC were from high-income countries (Australia, New Zealand, and USA) (Figure 1d). Reports of LC are common in Caucasians in Canada and Australia. More than half of the oral cancer cases in Australia can be found on the lip, and proximity of New Zealand could also explain the current results (Sugerman and Savage, 2002). Whereas in the USA, a case-control study done in California found solar radiation as a major risk in the development of lip cancer (Pogoda and Preston-Martin, 1996).

The country that garnered the lowest search for MC (47%) was the Philippines, while India ranked last in the web searches for TC (21%), GC (3%), and LC (4%). Both countries are Asian countries and categorized by the World Bank as lower-middle income countries. More than 1, 000, 000 new cases of oral squamous cell carcinoma have been recorded per year and incidence rates have multiplied in the last ten years. Districts in India like Bhopal and Ahmedabad were reported to have the highest incidence of TC and MC. The same study stated that India, Sri Lanka, Bangladesh, and Pakistan are countries where oral cancer is the most common cancer in males (Warnakulasuriya, 2009). In Sri Lanka, oral cancer is still a major problem due to the increasing betel quid use, despite the decrease in smoking habits and the 100% free health services. A study found that the level of awareness of people in Sri Lanka towards oral cancer is below 50%. They identified mass media as a potent tool in the prevention of oral cancer development. However, the population at risk consists of people in the lower social strata that have no access to this information (Somathunga et al., 2021). 

Another salient feature of this study is the discovery that search trends for MC, TC, GC, and LC have an upward slope (Figure 2a-d). While the observed data of oral cancer-related searches from 2010 to 2019 gradually increased, a decrease in 2020 data can be observed (Figure 2e), and the forecasted trendlines for 2021 and 2022 are stationary (Figure 3a-d). Data from the UK also show an upward trend since 1989 at an average of 2.7% annual increase. This has been attributed to alcoholism and binge drinking (Hindle et al., 2000). This rising trend was also reported in the USA from 1974-2004, while similar incidence rate increases were seen in France and Japan (Warnakulasuriya, 2009). 

However, the current results show that there are abrupt declines in searches during the COVID-19 pandemic in 2020. Challenges in early diagnosis (Chumpitaz-Cerrate et al., 2021), economic consequences (Coulthard et al., 2020a; Coulthard et al., 2020b), and treatment delay (Varela-Centelles et al., 2021) could alter the health-seeking behavior of a population during the pandemic. Teledentistry could also decrease the RSV of oral cancer by changing the information source of the population from GT to dental healthcare professionals (Deshpande et al., 2021). The inherent comorbidities and under prioritization of cancer, in favor of patients infected with SARS-CoV-2, render patients with cancer at-risk for the virus. In India, deficiencies in healthcare professionals, substandard infrastructures for healthcare, and the relative unpreparedness against the existing contagion have exacerbated the conditions of patients. The formation of a COVID-19 task force, deferring of complex medical procedures, telemedicine, creation of triages, and the establishment of protocols were done to ease the cancer situation during the current pandemic. Meanwhile, in Pakistan, the cancer treatments are delayed due to economic reasons leading to the lack of PPEs, medications, and hospitals (Yadav et al., 2021). The existing and widespread infection of SARS-CoV-2 has exposed problems in the healthcare systems, especially in low- and middle-income countries. Delays in cancer screening, diagnosis, prevention, treatment, and the disruption of medication supply chains have been identified in Asian countries like Singapore, Myanmar, Malaysia, Japan, Iran, Indonesia, India, and Bangladesh. Moreover, shortages of medical staff were reported in nine institutions (Singapore, Pakistan, Nepal, Myanmar, Malaysia, Iran, Indonesia, India, and Bangladesh). Prevention of cancer in the present and in preparation for upcoming pandemics, should be directed at restructuring redundant clinical and research requirements, publication of exploratory findings, heightening cooperation between cancer centers, and improvement of public cancer awareness (Gatellier et al., 2021).

*Study Limitations*

Although 250 countries and territories were considered, only 29 yielded the minimum data that could be calculated. The use of GT also limits the current study to RSV and not actual data that could show the demographics of the research participants. Moreover, aside from GT data, text from social media posts from Twitter and Facebook, or multimedia sources from YouTube and Instagram can also be mined for data. However, despite the limitations of this study, the results show certain similarities to other epidemiologic studies.

In summary, the data suggest that higher-income countries tend to be more interested in seeking information about oral cancer, although there were some exceptions (Philippines and Malaysia). The RSV for these searches had an upward trajectory from 2010 to 2019, which rapidly declined in 2020. In addition, the search for GC and LC, but not for TC, were significantly lower than that of MC. According to the forecasted data for 2021-2022, the peak popularity for all oral cancer-related search terms will not rise above the highest RSV of the 2010-2020 timeline.

## Author Contribution Statement

Junhel Dalanon: Conceptualization, Methodology, Formal Analysis, Investigation, Data Curation, Writing-Original Draft, Writing-Review & Editing, Visualization. Yoshizo Matsuka: Validation, Formal Analysis, Investigation, Resources, Data Curation, Writing-Review & Editing, Supervision, Project Administration, Funding Acquisition.
